# Targeted Therapies in Epithelial Ovarian Cancer

**DOI:** 10.1155/2010/314326

**Published:** 2010-01-13

**Authors:** Nicanor I. Barrena Medel, Jason D. Wright, Thomas J. Herzog

**Affiliations:** ^1^Division of Gynecologic Oncology, Department of Obstetrics & Gynecology, Columbia University College of Physicians and Surgeons, New York, NY 10032, USA; ^2^Gynecologic Oncology Division, Herbert Irving Comprehensive Cancer Center, New York, NY 10032, USA; ^3^Gynecologic Oncology Unit, Department of Obstetrics & Gynecology, Hospital Sótero del Río, Santiago, Chile

## Abstract

Epithelial ovarian cancer remains a major women's health problem due to its high lethality. Despite great efforts to develop effective prevention and early detection strategies, most patients are still diagnosed at advanced stages of disease. This pattern of late presentation has resulted in significant challenges in terms of designing effective therapies to achieve long-term cure. One potential promising strategy is the application of targeted therapeutics that exploit a myriad of critical pathways involved in tumorigenesis and metastasis. This review examines three of the most provocative targeted therapies with current or future applicability in epithelial ovarian cancer.

## 1. Introduction

Ovarian cancer represents the sixth most common malignancy as well as the seventh leading cause of cancer-related death in women worldwide [1, 2]. In the USA, this neoplasm ranks second among gynecologic cancers, yet it is by far the most lethal one, accounting for more than 15,000 deaths annually [3]. One of the major reasons underlying this dismal prognosis is the fact that nearly 75% of cases are diagnosed at an advanced stage (i.e., tumor already spread beyond the ovary) [4, 5], despite great efforts to develop reliable screening and prevention strategies. 

 To date, advanced ovarian cancer management has predominantly consisted of surgery followed by chemotherapy consisting of a combination of platinums and taxanes. More recently, neoadjuvant chemotherapy, a therapeutic alternative traditionally reserved for those patients considered poor candidates for upfront surgery, has emerged as a potential first-line option [6]. Even though up to 80% of these patients will respond to initial treatment, most of them will subsequently recur [7]. Chemotherapy success rates after relapse range from 10% to 50%, depending on whether the tumor is platinum sensitive or resistant (i.e., a progression-free interval (PFI) following platinum-based first-line therapy of more or less than 6 months, resp.). Unfortunately, almost all responses are invariably transient. Thus, the 5-year overall survival (OS) for late-stage disease is approximately 45% [2]. 

 Since “nonspecific” therapies, namely, surgery, radiation, and conventional chemotherapy, have largely failed to achieve cure in the majority of patients affected by epithelial ovarian cancer, investigators have focused on developing novel treatment approaches. Many of these new strategies are based upon an understanding of the critical molecules and pathways specifically involved in tumorigenesis and metastasis. This has led to the development of “targeted” oncologic therapies that might be ultimately more effective and less toxic.

 Although significant overlap occurs, targeted therapies can be broadly divided into two categories:

- those focused on cellular mechanisms that are disregulated in carcinogenesis,
- those directed against the neoplasm's microenvironment, a tumor component lately recognized as highly relevant in both cancer growth and dissemination.

The present article addresses targeted therapies currently being employed or tested in epithelial ovarian cancer (EOC). Since their number has become as numerous as the myriad of critical pathways involved in ovarian neoplastic transformation, this review will focus on three of the most promising and/or well-studied targeted weapons in ovarian cancer therapeutics to date, namely,

- antiangiogenesis compounds,
- epidermal growth factor receptor (EGFR) antagonists,
- poly (ADP) ribose polymerase (PARP) inhibitors.

## 2. Materials and Methods

A comprehensive literature search was conducted using the following key terms: “ovarian cancer”, “targeted therapies”, “antiangiogenesis”, “epidermal growth factor receptor (EGFR) inhibitors”, and “poly (ADP) ribose polymerase (PARP) inhibitors”. For this purpose, primary sources used were PubMed and Cochrane Databases. Articles' selection was limited to those written in English, without restriction to year of publication. The main analysis was focused on those studies providing clinical evidence, although preclinical data were included either when background information was required or when clinical assays were absent. Highly valuable references cited by primarily collected studies as well as pivotal abstracts presented at prominent oncologic meetings, such as the Society of Gynecologic Oncologists (SGO), the American Society of Clinical Oncology (ASCO), the European Society of Gynaecological Oncology (ESGO), and the International Gynecologic Cancer Society (IGCS), were also assessed and their data incorporated whenever pertinent.

## 3. Antiangiogenesis

Angiogenesis (i.e., the formation of new blood vessels) plays a critical role in cancer expansion and propagation. While many tumors start as avascular nodules, early data demonstrated that growth is impaired beyond 2 mm^3^ unless effective neovascularization is established [8]. Hence, this phenomenon appears to be a rate-limiting step in tumor progression. Antiangiogenic therapies have been shown to inhibit new blood vessels development, induce endothelial cells apoptosis, and normalize vasculature [5]. 

 Many components interact in this process, such as proangiogenic factors, metalloproteinases, and endothelial precursor cells. Among angiogenesis-promoting molecules, the vascular endothelial growth factor (VEGF) is the most sensitive and potent one, as well as the best characterized [9]. It is overexpressed in many human tumors, including ovarian cancer. In ovarian malignancies, high levels of VEGF have been associated with poor prognostic features, such as advanced stage, carcinomatosis, distant metastasis, as well as a decreased survival [10]. Thus, the VEGF pathway has become one of the most attractive research areas in EOC therapeutics. Preclinical data from animal models showed that VEGF blockade was associated with inhibition of ascites formation and tumor growth [11].

 Bevacizumab, a recombinant humanized monoclonal antibody directed against VEGF-A, was the first of these agents to be evaluated in EOC. Case reports and small series constituted the initial clinical evidence supporting its therapeutic value, mainly in recurrent, heavily pretreated patients [12, 13]. Based on these findings, two phase II trials using single-agent bevacizumab in recurrent ovarian cancer, predominantly platinum-resistant disease, were subsequently conducted (Table 1) [14, 15]. Their results demonstrated the following.

- An overall response rate (RR) was of 15%–21%. Unfortunately, less than 5% were complete responders.
- One study showed that additionally 50% of patients had stable disease.
- A 6-month progression-free survival (PFS) ranged from 30% to 40%.
- Hypertension was the most common side effect documented, being usually well controlled with standard antihypertensive medication. However, two major complications emerged, gastrointestinal perforation and thromboembolic disease, both venous and arterial, ranging from 0% to 11% and 3% to 7%, respectively. Indeed, one of the studies, Cannistra et al. [15], carried out in heavily pretreated patients, was prematurely closed due to the high incidence of bowel perforation observed.
- Bevacizumab-related deaths were estimated in up to 7% of treated patients.

This drug has been and continues to be tested in combination with chemotherapy, as a part of the first line treatment in newly diagnosed EOC and recurrent disease. Table 1 both summarizes the most relevant past and ongoing trials conducted in this setting.

 Other anti-VEGF as well as non-VEGF mediated Antiangiogenic drugs are currently in clinical development. Table 2 illustrates some of these initiatives.

 In conclusion, to date antiangiogenesis appears as one of the most promising targeted strategies explored in EOC. Given the encouraging initial results, bevacizumab has entered phase III trial evaluation. Meanwhile, it is considered a viable option in the recurrent setting. Appropiate bevacizumab dose (7.5 versus 15 mg/kg) and the ability to combine with other biologics require further study as well. Safety issues must be considered when using this compound. Adequate patient selection may potentially reduce the incidence of serious adverse events by excluding those at a highest risk for gastrointestinal perforation or a thromboembolic event. Major risk factors for these two complications have been described (Table 3), yet it should be noted that they still require further validation. 

## 4. Epidermal Growth Factor (EGF) Receptors Antagonists

The family of EGF receptors (EGFRs) is composed of 4 structurally similar receptors which exert a tyrosine kinase function: ErbB1 (commonly referred to as epidermal growth factor receptor), ErbB2 (Her2/neu), ErbB3 (Her3), and ErbB4 (Her4). Their activation triggers a cascade of events ultimately resulting in cell proliferation and survival. Like VEGF, EGFRs are frequently overexpressed and/or dysregulated in solid tumors. Ovarian cancer is not an exception, with up to 70% of cases exhibiting this aberrant phenotype, which has been linked to poor oncologic features and outcomes [5, 7, 29].

 These observations have suggested that EGFRs might represent a viable target for novel therapies in EOC. While blockade of these receptors can be achieved by several mechanisms, two of these have been most extensively explored: (a) small molecules capable of inhibiting the tyrosine kinase domain and (b) monoclonal antibodies directed against the extracellular region. 

 Preliminary preclinical data demonstrated antitumoral activity and a reversion of the chemoresistant phenotype secondary to EGFR inhibition [30]. Nonetheless, and in contrast with the promising results obtained with anti-VEGF therapies, to date clinical trials with EGFR inhibitors alone have produced disappointing results. 

### 4.1. Tyrosine Kinase Inhibitors (TKIs)

Gefitinib and erlotinib are two of the main compounds in this category. Both are orally administrated and relatively well tolerated [31], which would represent a significant advantage in terms of patients' quality of life. Core findings from the most relevant trials conducted on these agents can be summarized as follows [32–38].

- An RR in recurrent ovarian cancer is of less than 10% along with stable disease in up to 44% of patients when used as single agents.
- These results were improved either when gefitinib was combined with standard chemotherapy or when erlotinib was combined with bevacizumab. The combination gefitinib-tamoxifen did not appear to add any clinical benefit.
- As a part of the first-line treatment in conjunction with a platinum and a taxane, either upfront or as consolidation therapy, TKIs have yet to confirm a demonstrable survival advantage. The EORTC has just finalized the recruitment of a phase III trial exploring erlotinib as maintenance therapy in both high-risk early-stage and advanced diseases.
- In terms of side effects, the most frequently observed were diarrhea (up to 30%, being the dose-limiting toxicity), nauseas and vomiting (nearly 10%), and an acne-like cutaneous rash (5%–15%), which interestingly correlated positively with tumor response. As expected, increased toxicity was seen when a cytotoxic agent was coadministrated.

As noted above, the initial experience with these agents has not revealed a definitive role in the treatment of unselected EOC population, either in the first-line or the relapsed setting. However, a subgroup of patients showing an increased likelihood to respond to these compounds has been identified. In Schilder's trial, published in 2005, clinical outcomes correlated with EGFR status, with a significantly longer progression-free survival (PFS) as well as a trend in improved overall survival (OS) among those who were EGFR (+). Specifically, an enhanced response to gefitinib was linked to the presence of an infrequent mutation affecting the catalytic domain of this receptor [32]. This relationship closely resembles what previously has been described in lung cancer [39, 40]. Thus, it has been suggested that prescreening patients for specific active EGFR mutations could define the population most likely to benefit from this therapy. Further investigation to validate this finding in EOC is warranted. 

 Novel EGFR inhibitors in development for EOC include lapatinib, canertinib, PKI-166, and EKB-569. Until better evidence supporting a relevant therapeutic value becomes available, the role of TKIs in this neoplasm remains predominately confined to clinical trials.

### 4.2. Monoclonal Antibodies

Various humanized antibodies against the extracellular region of EGFR have been thought to be potentially effective in EOC. Nonetheless, similar to what has occurred with TKIs, the theory has not been confirmed clinically. Probably the most emblematic example illustrating this unfulfilled potential has been trastuzumab. Multiple initial studies confirmed that Her-2/neu overexpression was associated with an adverse prognosis of patients with epithelial ovarian cancer [41, 42]. Trastuzumab, a selective Her-2/neu inhibitor approved for the treatment of ErbB 2 (+) metastatic breast cancer, was proposed to have antitumoral activity comensurate with that observed in breast cancer. Further clinical evidence in a large GOG trial, however, demonstrated a response rate of only 7%, with disease stabilization in other 39% of ErbB 2(+) recurrent ovarian cancer patients [43]. 

 Results obtained with other monoclonal antibodies, alone or in combination with standard chemotherapy, are outlined in Table 4.

## 5. PARP Inhibitors

Approximately 10% of ovarian cancers are considered hereditary. Germline mutations affecting two genes, BRCA1 and BRCA2, account for the vast majority of these cases. The lifetime risk of developing an epithelial ovarian carcinoma among women who carry these genetic defects has been estimated to be up to 60% [49]. The proteins encoded by these tumor suppressor genes participate in multiple cellular processes, including transcription, cell cycle regulation, and repair of DNA double-strand breaks [50]. When inactived, chromosome instability occurs, an event potentially facilitating carcinogenesis. 

 Many other DNA-repair mechanisms are generally available within the normal cell. The base-excision repair (BER) complex constitutes one of them. The enzyme poly (ADP) ribose polymerase (PARP) is a key component of this pathway. Its scope is restricted to single-strand defects. Accordingly, its malfunction theoretically should not affect double-strand repair; however, a persistent single-strand defect may ultimately result in DNA replication interruption or a double-strand break [51]. When this occurs in a cell that is already unable to repair DNA damage , as the case for BRCA-defective cells, cell cycle arrest or death occurs. This observation, known as synthetic lethality [52], supports the contention that PARP blockade would be therapeutically effective in hereditary EOC. This premise was initially confirmed in preclinical studies demonstrating a highly increased sensitivity to PARP inhibition among BRCA-deficient cells, with a subsequent decreased cell survival, compared to those heterozygous or BRCA wild-type cells [53, 54]. 

 Clinical studies exploiting this approach have been recently conducted in multiple human solid tumors. Initial trials used these agents primarily as chemosensitizers, mainly in association with methylating compounds [55]. However, with the demonstration of BRCA specific sensitivity, single-agent inhibitors were assessed. Recently, final results of the first phase I trial evaluating Olaparib, an orally administrated PARP inhibitor, in BRCA-defective malignancies, including ovarian cancer, showed a low toxicity along with a response or disease stabilization rate of 63% [56]. Multiple PARP inhibitors are currently being examined in phase II trials. Of interest, Audeh et al. lately reported the interim analysis of a phase II study employing Olaparib in BRCA-deficient advanced ovarian cancer [57]. Overall 57% of patients demonstrated response to PARP inhibition, using either RECIST or CA-125 criteria. Potential use of PARP inhibitors as chemoprophylactics in BRCA mutation carriers [58] and for treating sporadic ovarian cancers [49] has been proposed, as well. 

 A potential barrier to PARP inhibitors use has been the recently described emergence of resistance by reversal of the BRCA-deficient phenotype [59]. The clinical implications of this phenomenon require further clarification.

## 6. Conclusions and Future Overview

Women with epithelial ovarian cancer are living for longer periods of time than ever before. Development of novel chemotherapeutics has in part contributed to this improved outcome. However, a significant proportion of affected patients still succumbs to this difficult disease. Thus, progress is still needed. To this end, targeted therapies appear to be a promising platform for clinical development.

 Many cellular pathways have been implicated in ovarian carcinogenesis, and exploitation of these perturbations critical in forming or maintaining the malignant phenotype has yielded a number of promising compounds. However, to date only Antiangiogenic agents have reached clinical relevance in EOC management. New therapeutic tools showing promising results, such as PARP inhibitors that exploit the abnormality responsible for the initial neoplastic transformation, have demonstrated encouraging clinical potential. 

 Some relevant lessons learned in targeted therapy development thus far include [7] the following.

- The mere presence of a particular molecule or pathway dysregulated in a particular tumor does not guarantee that its inactivation will have therapeutic benefit.
- Response does not always translate into prolonged survival, symptom relief, or other valuable clinical endpoints. Conversely, there may be significant improvements in time-to-event endpoints such as time-to-progression or PFS, and yet objective responses may be rare. Thus different clinical parameters may be neccesary for efficacy assessment of targeted agents.
- Given the multiplicity and redundancy of aberrant pathways involved in ovarian cancer, it is unlikely that inhibition of a single cascade will be highly effective. Thus agents that act upon multiple levels or interconnected pathways simultaneously appear potentially more promising.

The future of cancer therapeutics will likely include tailored, individualized treatments, designed on the basis of an even deeper understanding of the critical alterations in ovarian carcinogenesis. Gene expression profiles have established that this neoplasia is far from being a uniform disease [60]. Thus, genotype-directed and pharmacogenomic therapies emerge as the next frontier for fruitful exploration and novel drug development.

## Figures and Tables

**Table 1:** Clinical trials testing Bevacizumab in EOC.

	Type	Study's Scope and Population	Intervention	Outcomes or Planned End Points
Published				

Burger (2007) [14]	Phase II			CR: 3%
		62 patients with persistent or recurrent Ov or PP cancers		PR: 18%
				SD: 52%
		66% had received two prior chemotherapy regimens	Single-agent Bevacizumab	MPFS: 4.7
				6-mon PFS: 40%
		42% were platinum-resistant		MOS: 17
				GIP: 0%
				TED: 0%

Cannistra (*) (2007) [15]	Phase II			CR: 0%
		44 patients with recurrent Ov or PP cancers		PR: 16%
		48% had received three prior chemotherapy regimens	Single-agent Bevacizumab	SD: Not reported
				MPFS: 4.4
		84% were platinum-resistant		MOS: 10.7
				GIP: 11%
				TED: 7%

Micha (2007) [16]	Phase II			CR:30%
		Adjuvant treatment in front-line	Carboplatin + Paclitaxel + Bevacizumab	PR:50%
		20 patients stage III Ov, PP, or FT cancers		SD: 5%
		85% optimally cytoreduced		TED: 10% (**)
				GIP: 0%

Garcia (2008) [17]	Phase II			CR: 0%
				PR: 24%
		70 patients with recurrent Ov or PP cancers	Metronomic Cyclophosphamide + Bevacizumab	SD: 63%
		Median n^*º*^ of prior chemotherapy regimens: 2		MOS: 17
		40% were platinum-resistant		MPFS: 7
				TED: 4%
				GIP: 4%

Ongoing				

TEACO	Phase II			1-year PFS
		Adjuvant treatment in front-line	Oxaliplatin + Docetaxel + Bevacizumab (both first line and maintenance)	Safety
		Stage IB-IV Ov, PP, or FT cancers		RR
		Either optimally or suboptimally cytoreduced		PFS
				OS

GOG 218	Phase III			PFS (primary)
		Adjuvant treatment in front-line	Carboplatin + Paclitaxel with or without Bevacizumab, either short-term or extended (maintenance)	OS
		Stage III-IV Ov or PP cancers		RR
		Either optimally or suboptimally cytoreduced		Toxicity
				QoL
				Translational objectives

ICON 7	Phase III	Adjuvant treatment in front-line		PFS (primary)
		High-risk early stage (I-IIA, clear cell or grade 3) or advanced stage (IIB or greater), either optimally or suboptimally cytoreduced Ov, PP, or FT cancers	Carboplatin + Paclitaxel with or without extended Bevacizumab	QoL
				Cost effectiveness

GOG 213	Phase III	Platinum-sensitive recurrent Ov, PP, or FT cancers	Carboplatin + Taxane with or without Bevacizumab with or without Secondary cytoreduction	OS (primary)
				PFS
				Toxicity

OCEANS (AVF4095g)	Phase III	Platinum-sensitive recurrent Ov, PP, or FT cancers	Carboplatin + Gemcitabine with or without both short-and long-term (manitenance) Bevacizumab	PFS
				OS
				RR
				Safety profile of the combination

Ov: Ovarian; PP: Primary peritoneal; FT: Fallopian Tube

RR: Response rate; CR: Complete response; PR: Partial response; SD: Stable disease

TED: Thromboembolic disease (either arterial or venous); GIP: Gastrointestinal perforation

MPFS: Median progression-free survival (months); MOS: Median overall survival (months);

QoL: Quality of life

(*) Study stopped prematurely due to the high rate of severe complications (i.e., GIP)

(**) TED cases were not directly attributed to bevacizumab.

**Table 2:** Examples of other promising Antiangiogenic agents in EOC.

	Mechanism of action	Current evidence
VEGF-mediated		

Aflibercept (VEGF-Trap)	Soluble receptor which binds VEGF-A and-B as well as placenta-derived growth factor (PlGF) 1 and 2	Preliminary results reported by a Phase II trial conducted in recurrent setting showed similar results than bevacizumab, with a remarkable less incidence of bowel perforation (1%) [18]
		A phase III trial is ongoing

Cediranib	Small molecule that inhibits the tyrosine kinase domain of the VEFG receptor (VEGFR)	Two phase II trials in relapsing EOC demonstrated a response rate of nearly 20%, increasing up to 30% if disease stabilization is considered [19, 20]
	Other members of this family are sorafenib and sunitinib	ICON 6, a phase III trial in recurrent platinum-sensitive patients, is now testing this agent in combination with carboplatin and paclitaxel

Sorafenib	Multitargeted TKI that inhibits raf kinase, VEGFR-2, VEGFR-3, Flt-3, c-kit, and platelet-derived growth factor receptor (PDGFR)	Phase I trial reported that 50% of patients showed stable disease [21]. Early data from a subsequent phase II study testing the combination of sorafenib with gemcitabine in recurrent EOC confirmed encouraging activity, with an overall response rate of 33% [22]
		Several other phase II trials employing sorafenib either in front-line, maintenance phase, or recurrent settings, alone or in combination with standard chemotherapy or biologics (e.g., bevacizumab) are underway
		A randomized phase III trial is currently evaluating Sorafenib as a maintenance therapy after first-line treatment in EOC

Pazopanib	Oral tyrosine kinase inhibitor that targets VEGFR, PDGFR, and c-kit	Preliminary results of a phase II trial conducted in recurrent EOC defined by CA-125 elevation showed a biochemical response of 47%, with stable disease observed in other 27% [23]
		A phase III trial is currently evaluating pazopanib as a maintenance therapy after first-line treatment in EOC

Non VEGF-mediated		

Vascular disrupting agents (VDAs)	Represent a new approach to deprive tumor from its blood supply, by causing the collapse of the established tumor vasculature. Their main targets are the endothelial cells	Preclinical data indicate that these drugs can improve tumor response to chemotherapy [24], radiation, and other Antiangiogenic therapies
	Examples include tubulin destabilizers and flavanoids, among others	Zweifel and coworkers presented recently the final results of a phase II trial employing Fosbretabulin (a tubulin binder) along with carboplatin and paclitaxel in platinum-resistant EOC, revealing a response rate of 32% [25]

**Table 3:** Major risks factors potentially associated with bevacizumab-induced arterial thrombo-embolism and gastrointestinal perforation.

1- Arterial Thromboembolic Events (ATEs) [26]
–Age ≥65 years
–Prior history of ATE
2- Gastrointestinal Perforation [27, 28]
–Multiple prior chemotherapy regimens (heavily pretreated patients)
–Large intraabdominal tumor burden
–Neoplastic bowel involvement
–Clinical evidence of partial obstruction

**Table 4:** Other Anti-EGFR antibodies explored in EOC.

Antibody	Target	Clinical data available
Cetuximab	ErbB1	Three phase II trials (one as a component of the first-line treatment and two performed in recurrence), alone or in combination with conventional cytotoxic therapy, have evidenced null or only modest impact of cetuximab in the management of EOC [44–46]

Matuzumab	ErbB1	One phase II study conducted in platinum-resistant, EGFR (+) population, concluded that matuzumab was well-tolerated, but lacked significant clinical activity [47]

Pertuzumab	Her2/neu	One phase II trial involving advanced, refractory EOC patients has been conducted using this agent. Like matuzumab, pertuzumab was associated with a poor response rate (approximately 4%) [48]
